# Knowledge, attitude, and practice of dental professionals towards green dentistry in Karachi city- a cross-sectional survey

**DOI:** 10.1186/s12903-025-07215-4

**Published:** 2025-11-24

**Authors:** Muhammad Sadiq Billoo, Shahrukh Ali Khan, Syed Murtaza Raza Kazmi, Farah Jabeen, Taimur Khalid

**Affiliations:** 1 Section of Prosthodontics, Department of Dentistry and Oral Health Sciences, Aga Khan University Hospital, P.O. Box 3500, JHS Building 1st- Floor Dental Clinics, Stadium Road, Karachi, 74800 Pakistan; 2 Department of Prosthodontics, Fatima Jinnah Dental College, University of Karachi, Karachi, Pakistan; 3 AKDN dHRC Aga Khan University, Karachi, Pakistan

**Keywords:** Eco-friendly dentistry, Green dentistry, Sustainable dentistry, Recycle

## Abstract

**Introduction:**

Environmental degradation from pollution, global warming, and industrialization, is alarming with dentistry significantly contributing through mercury and plastic toxicity from improper waste handling.

**Aim:**

To evaluate the knowledge, attitude, and practice of Dental professionals towards “Green Dentistry” working in the public and private sectors of Karachi city.

**Design:**

A cross-sectional survey.

**Setting:**

Karachi, Pakistan (Online).

**Materials and methods:**

An online questionnaire was conducted among dentists of Karachi, Pakistan, from January to June 2023 collecting participant’s demographic data and assessing knowledge, attitude, and practice of eco-friendly dentistry.

**Results:**

With a 61% response rate, most respondents (48.5%) were General Dental Practitioners (GDPs). Dental consultants (DC) exhibited higher awareness of green dentistry (29%) with 50% of DCs favoring digital radiography as environmentally friendly (*p* < 0.05). Green dentistry practices were more prevalent among DCs with 42.8% (*p* < 0.05) eliminating amalgam usage, while more GDPs (72.2%, *p* < 0.05) adopted re-usable lab coats and patient drapes.

**Discussion and conclusion:**

Awareness of “Green Dentistry” is generally lacking among participants. Both GDPs and DCs are inclined towards adopting environment-friendly dental practices. Education on energy conservation and materials is essential to reduce waste in the dental industry.

**Supplementary Information:**

The online version contains supplementary material available at 10.1186/s12903-025-07215-4.

## Introduction

While industrialization has significantly improved the quality of life, it has also contributed to deforestation, global warming, and pollution [1]. Dentistry, though essential for oral health, plays a role in environmental degradation through the mismanagement of mercury, plastics, and various biological or synthetic wastes [2]. These waste products include biological materials (e.g., extracted teeth, biopsy tissue), hazardous substances (e.g., mercury from amalgam), chemical agents (e.g., X-ray film developers, instrument lubricants) and disposable items (e.g., latex gloves, plastic suction tips [3, 4]. All these wastes collectively form a majority of dental wastes in the dental clinic setting [5]. The potential harm caused by improper handling of these materials is a growing concern, prompting environmentalists and policymakers to seek immediate sustainable solutions.

To mitigate the environmental impact of dental practices, the Eco Dentistry Association (EDA) has introduced the concept of “Green Dentistry” [6]. It is defined as “A high-tech approach that reduces the environmental impact of dental practices and encompasses a service model for dentistry that supports and maintains wellness” [2]. It aims to minimize waste and energy consumption, ultimately benefiting both the environmental and dental professions. This approach promotes the adoption of advanced technologies like digital radiography and intra-oral scanning, reducing the need for physical materials, thus averting waste production [7]. To facilitate this revolution in dentistry, the concept of “Four Rs” (Rethink, Reduce, Reuse, and Recycle) has previously been recommended [8]. ‘Rethinking’ is crucial to minimizing any unnecessary waste production, thereby averting further harm to the environment. ‘Reduce’ focuses on minimizing excess waste and reducing water consumption. ‘Re-use’ emphasizes the repeated use of products, including hand towels, biodegradable cups instead of plastic ones, and sterilizable instruments. ‘Recycling’ refers to converting used materials into reusable items, such as recycling paper cups and using rechargeable products.

Although green dentistry is gaining traction in developed countries, its adoption in South Asian countries like India [6, 9], and Thailand [10], remains limited. In Pakistan, there haven’t been any notable initiatives yet to promote the adoption of Green Dentistry, which can be owed to a lack of policy reinforcement and institutional preferences as highlighted elsewhere, despite attempts to introduce environmental friendly initiatives [11]. Therefore, this study aims to assess dentists’ knowledge, attitude and practice towards implementing environmentally friendly practices in a local setting, among dental consultants (dentists who have undergone recognized postgraduate specialty training of approximately 3 to 5 years following their primary dental qualification), post-graduate residents (dentists currently undergoing specialty training) and general dental practitioners. The findings are intended to inform strategies that can reduce material and energy waste through increased awareness and institutional support.

## Materials and methods

This web-based, cross-sectional analytical survey was conducted across multiple centers and included dentists from both public and private sectors in Pakistan. The sample size was calculated using WHO software, based on the hypothesis that at least 280 dentists from Karachi would be aware of green dentistry. The calculation assumed a power of 90% to detect a 0.09 difference in green dentistry awareness [6], employing a two-sided test at a significance level of 0.05.

The questionnaire (Annexure I) was developed after reviewing relevant literature [10, 12, 13]. The instrument was pre-tested on a pilot group of 30 participants to evaluate understanding by the participants and to identify and revise any ambiguous items that could cause confusion. Since no discrepancies were identified and the pretested participants met the same inclusion criteria as the main sample, their data were included in the final analysis in line with recommendations on incorporating pretest data when no changes are required in the final analysis [14]. 

To establish content validity, five subject experts reviewed the instrument. The Scale-level Content Validity Index (S-CVI) scores were 1.00 for knowledge, 0.96 for attitude, and 0.98 for practice, indicating strong expert agreement. Item-level CVI (I-CVI) values also showed excellent agreement, with scores approaching 1.0. Internal consistency of the tool was assessed using Cronbach’s α. The knowledge domain demonstrated excellent reliability (α = 0.83), while the attitude domain showed acceptable reliability (α = 0.73). The practice domain produced a Cronbach’s α of 0.58, indicating moderate reliability. The acceptable threshold for Cronbach’s α from 0.4 to 0.7 was based on established literature [15].

A total of 280 Questionnaires were distributed over a six-month period beginning January 1, 2023. The study protocol received exempt approval from the Ethical Review Committee of the Aga Khan University Hospital, Karachi (ERC no. 2023-8554-24444), as no interventions were undertaken, and participation was anonymous.

Eligible participants included general dental practitioners (GDPs), postgraduate residents (PGRs), and dental consultants (DCs). Dental undergraduates, non-practicing dentists, and participants with incomplete survey forms were excluded. The self-administered questionnaire was developed using Google Forms and distributed electronically to participants whose valid email addresses were available. The survey began with an informed consent statement outlining the study’s purpose and ensuring confidentiality, with permission to use collective responses for publication purposes.

Demographic information collected included age, gender, alma mater, education level and current academic position. The knowledge segment assessed the overall awareness of “Green Dentistry” concepts among dental professionals. The attitude section probed participants’ perspectives on the impact and practicality of adopting green practices within dental clinics. Meanwhile, the practice section focused on questions related to the four ‘Rs’ of eco-friendly dentistry. Data was analyzed using STATA 16.0. The frequencies and proportions of the descriptive variables were reported. Chi-square tests were used to examine associations, with a 95% confidence interval and statistical significance set at *p* < 0.05. Logistic regression was performed to explore associations between knowledge, attitude and practice of Green dentistry.

## Results

**Fig. 1:**
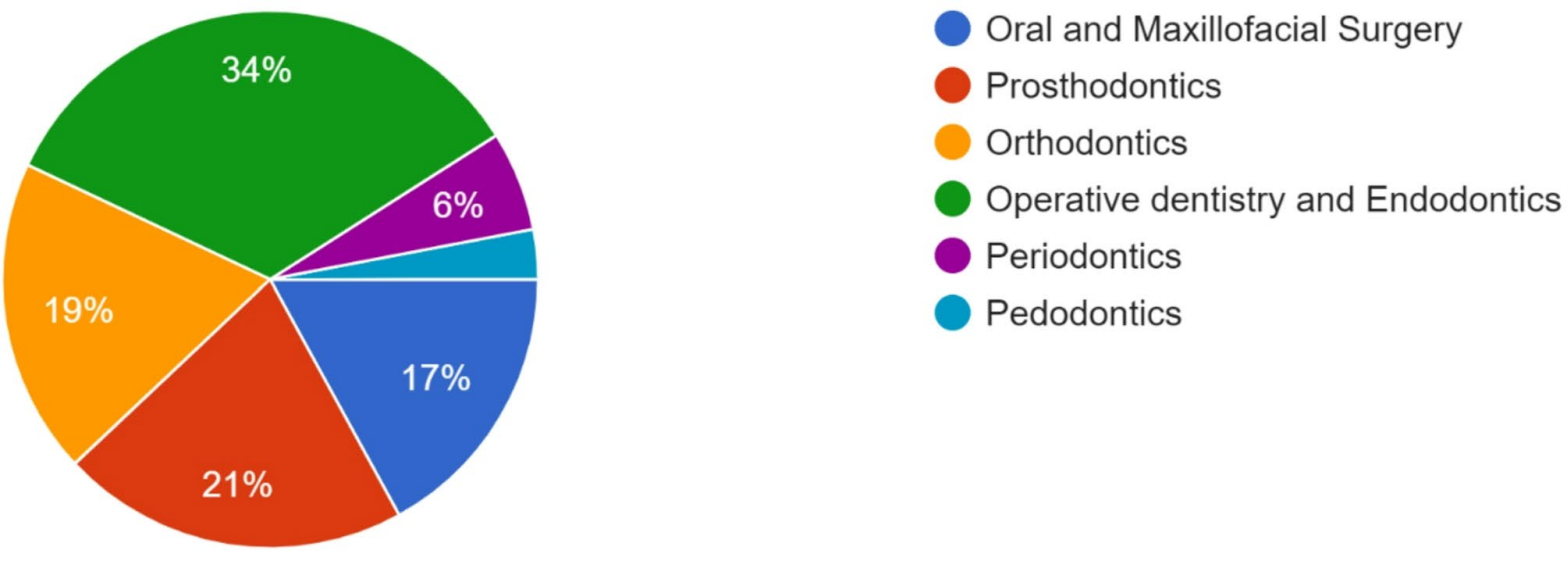
Distribution of dental specialities (*n* = 42) contribution among a sample of Pakistani dentists (*N* = 171)

A 61% response rate was obtained from 171 out of 280 participants. The sample included 103 females (60.2%) and 68 males (39.8%). The mean age of participants was 30.7 ± 7.4, reflecting a relatively young cohort. In terms of professional roles, general dental practitioners (GDPs) made up the largest group (*n* = 83/171; 48.5%), followed by postgraduate residents (PGRs) (*n* = 46/171; 26.9%) and dental consultants (DCs) (*n* = 42/171; 24.5%). Over half of the respondents (58.48%, *n* = 100) graduated from private institutions, while 41.5% (*n* = 71) attended public colleges. The distribution of dental specializations among the respondents is depicted in Fig. 1.

Most participants (*n* = 111; 65%) lacked a clear understanding of green dentistry. This was consistent across all groups: 55% of DCs (*n* = 23), 66% of GDPs (*n* = 55) and 72% of PGRs (*n* = 33), with no significant group differences (*p* = 0.23). Similarly, familiarity with the “4Rs” of Green Dentistry was low and comparable among groups; 29% of DCs (*n* = 12), 27% of GDPs (*n* = 22), and 22% of PGRs (*n* = 10) with no significant difference (*p* = 0.74).

Half (50%) of DCs, 39% of GDPs and 37% of PGRs recognized “green dentistry” and “eco-friendly dentistry” as interchangeable terms (*p* = 0.38). Awareness of “Sustainable dentistry” was reported by 43% of DCs, 37% of GDPs and 22% of PGRs, with no significant difference between the groups (*p* = 0.086). Awareness of the 2015 United Nations (UN) sustainable development agenda was limited across all the groups: 31% of DCs, 19% of GDPs, and 11% of PGRs.

Most attitude-related responses towards Green Dentistry showed no significant differences between groups (Table 1 and 2) (*p* > 0.05), except perceptions regarding digital radiography’s ecological benefits (*p* = 0.003). A higher proportion of GDPs (*n* = 76;91.6%) and PGRs (*n* = 37;80.4%) supported this compared to DCs (*n* = 36; 85.7%).

**Table 1:** Reverse attitude assessment of the included participants among a sample of Pakistani dentists (*N* = 171)

	Dental consultant	General Dental Practitioners	Postgraduate Residents	*P* -value
	*n* = 42	*n* = 83	*n* = 46	
	*n* (%)	*n* (%)	*n* (%)	
There is nothing a small sector dental practice can do for environmental issues				0.068
Strongly Agree	1(2.38)	3 (3.7)	0	
Agree	6 (14.29)	16 (19.75)	7 (15.22)	
Neutral	16 (38.1)	15 (18.52)	17 (36.96)	
Disagree	10 (23.81)	28 (34.57)	19 (41.3)	
Strongly Disagree	9 (21.43)	19 (23.46)	3 (6.52)	
It is difficult to change my current practice to be more ecological				0.077
Strongly Agree	1(2.38)	4 (4.82)	2 (4.35)	
Agree	6 (14.29)	19 (22.89)	15 (32.61)	
Neutral	17 (40.48)	32 (38.55)	22 (47.83)	
Disagree	13 (30.95)	25 (30.12)	7 (15.22)	
Strongly Disagree	5 (11.9)	3 (3.61)	0	
Creating a green dental clinic will increase the financial burden on management of the practice				0.381
Strongly Agree	3 (7.14)	4 (4.82)	3 (6.52)	
Agree	11 (26.19)	32 (38.55)	23 (50)	
Neutral	17 (40.48)	33 (39.76)	14 (30.43)	
Disagree	6 (14.29)	11 (13.25)	4 (8.7)	
Strongly Disagree	5 (11.9)	3 (3.61)	2 (4.35)	
It is difficult to find eco-friendly products for the dental clinic				0.397
Strongly Agree	2 (4.76)	4 (4.82)	3 (6.52)	
Agree	12 (28.57)	37 (44.58)	22 (47.83)	
Neutral	17 (40.48)	23 (27.71)	14 (30.43)	
Disagree	7 (16.67)	15 (18.07)	7 (15.22)	
Strongly Disagree	4 (9.52)	4 (4.82)	0	
Recycling waste management is an extra burden on the work of our practice				0.185
Strongly Agree	5 (11.9)	3 (3.61)	1 (2.17)	
Agree	11 (26.19)	33 (39.76)	16 (34.78)	
Neutral	14 (33.33)	27 (32.53)	20 (43.48)	
Disagree	7 (16.67)	16 (19.28)	8 (17.39)	
Strongly Disagree	5 (11.9)	4 (4.82)	1(2.17)	
Sterilized instruments are not as clean as disposable instruments				0.193
Strongly Agree	1 (2.38)	1(1.2)	1 (2.17)	
Agree	3 (7.14)	15 (18.07)	12 (26.09)	
Neutral	9 (21.43)	19 (22.89)	11 (23.91)	
Disagree	17 (40.48)	34 (40.96)	19 (41.3)	
Strongly Disagree	12 (28.57)	14 (16.87)	3 (6.52)	
It is not necessary to control water conservation				0.204
Strongly Agree	3 (7.14)	5 (6.02)	1 (2.17)	
Agree	1 (2.38)	10 (12.05)	5 (10.87)	
Neutral	8 (19.05)	8 (9.64)	9 (19.57)	
Disagree	15 (35.71)	30 (36.14)	22 (47.83)	
Strongly Disagree	15 (35.71)	30 (36.14)	9 (19.57)	

Significant* p*-values (≤ 0.05 denoted with “*”) are reported from Chi-square test

**Table 2:** Positive attitude assessment of the included participants among a sample of Pakistani dentists (*N* = 171)

	Dental consultant	General Dental Practitioners	Postgraduate Residents	*P* -value
	*n* = 42	*n* = 83	*n* = 46	
	*n* (%)	*n* (%)	*n* (%)	
Green practice has many long-term benefits (e.g., reduced energy and water costs)				0.439
Strongly Agree	11 (26.19)	25 (30.12)	6 (13.04)	
Agree	19 (45.24)	44 (53.01)	28 (60.87)	
Neutral	9 (21.43)	8 (9.64)	9 (19.57)	
Disagree	2 (4.76)	4 (4.82)	2 (4.35)	
Strongly Disagree	1 (2.38)	2 (2.41)	1 (2.17)	
Tooth colour-like materials are more environmentally friendly than amalgam				0.615
Strongly Agree	17 (40.48)	22 (26.83)	9 (20)	
Agree	18 (42.86)	46 (56.1)	28 (62.22)	
Neutral	6 (14.29)	12 (14.63)	7 (15.56)	
Disagree	0	1 (1.22)	0	
Strongly Disagree	1 (2.38)	1 (1.22)	1 (2.22)	
Digital radiography is more ecological than conventional techniques				0.003*
Strongly Agree	21 (50)	35 (42.17)	7 (15.22)	
Agree	15 (35.71)	41 (49.4)	30 (65.22)	
Neutral	4 (9.52)	4 (4.82)	5 (10.87)	
Disagree	0	1 (1.2)	4 (8.7)	
Strongly Disagree	2 (4.76)	2 (2.41)	0	
Energy management is everyone’s responsibility				0.652
Strongly Agree	14 (33.33)	35 (42.17)	14 (30.43)	
Agree	21 (50)	37 (44.58)	26 (56.52)	
Neutral	6 (14.29)	8 (9.64)	6 (13.04)	
Disagree	0	2 (2.41)	0	
Strongly Disagree	1(2.38)	1(1.2)	0	

Significant* p*-values (≤ 0.05 denoted with “*”) are reported from Chi-square test

While not statistically significant, a higher proportion of GDPs (*n* = 69;83.13%), PGRs (*n* = 34; 74%) and DCs (*n* = 31; 71.4%) agreed on the long-term benefits of green practices, such as reduced utility costs (*p =* 0.439). Opinions regarding the availability of eco-friendly products were mixed across all groups, without statistical significance (*p* = 0.397).

Regarding practice assessment (Table 3), two significant differences were identified. First, the frequency of amalgam restoration use varied significantly (= 0.02), with 30.4% of PGRs(*n* = 14) using amalgam “sometimes” compared to 16.7% of DCs (*n* = 7) and 13.3% of GDPs (*n* = 11). Second, the use of re-usable lab coats and patient drapes differed significantly (*p* = 0.048); 72.3% of GDPs, 63.04% of PGRs utilized more reusable *p* items compared to only 50% of DCs. Additionally, there were no significant differences among groups in the use of digital dentistry technologies, such as digital impressions, digital patient charting, and CAD/CAM systems.

**Table 3:** Practice assessment including four Rs of green dentistry of the included participants among a sample of Pakistani dentists (*N* = 171)

	Dental consultants	General Dental Practitioners (GDPs)	Postgraduate Residents	*p* -value
	*n* = 42	*n* = 83	*n* = 46	
	*n* (%)	*n* (%)	*n* (%)	
*Rethink*				
Lighting system used in your practice				0.513
LED lightbulbs with motion sensors	24 (57.14)	45 (54.22)	24 (52.17)	
Compact fluorescent lamps (CFL)	6 (14.29)	7 (8.43)	6 (13.04)	
Incandescent bulbs	3 (7.14)	15 (18.07)	4 (8.7)	
Other	9 (21.43)	16 (19.28)	12 (26.09)	
Should we go digital to eliminate photochemical waste?				0.292
Yes	37 (88.1)	79 (95.18)	41 (89.13)	
No	5 (11.9)	4 (4.82)	5 (10.87)	
Should Eco-friendly dentistry be universally recommended?				0.761
Yes	38 (90.48)	78 (93.98)	43 (93.48)	
No	4 (9.52)	5 (6.02)	3 (6.52)	
*Reduce*				
Use composite and/or GIC as an alternative to amalgam restorations.				0.402
Yes	38 (90.48)	80 (96.39)	43 (93.48)	
No	4 (9.52)	3 (3.61)	3 (6.52)	
Frequency of amalgam restorations performed in the clinic				0.02*
Always	0 (0)	2 (2.41)	0 (0)	
Often	6 (14.29)	11 (13.25)	8 (17.39)	
Sometimes	7 (16.67)	11 (13.25)	14 (30.43)	
Rarely	11 (26.19)	26 (31.33)	19 (41.3)	
Never	18 (42.86)	33 (39.76)	5 (10.87)	
Availability of an amalgamator in the clinic				0.458
Yes	18 (42.86)	34 (40.96)	24 (52.17)	
No	24 (57.14)	49 (59.04)	22 (47.83)	
Method of mercury disposal				0.089
In Sewerage system	10 (23.81)	9 (10.84)	5 (10.87)	
In clinical waste bags or sharp containers	22 (52.38)	48 (57.83)	20 (43.48)	
In waste destined for incineration	8 (19.05)	19 (22.89)	19 (41.3)	
Recycle through Amalgam separator	2 (4.76)	7 (8.43)	2 (4.35)	
*Re-use*				
Use reusable sterilization instruments (e.g., trays, film holders) instead of disposables				0.706
Yes	35 (83.33)	64 (77.11)	37 (80.43)	
No	7 (16.67)	19 (22.89)	9 (19.57)	
Use disposable plastic instead of reusable glass irrigation syringes				0.512
Yes	35 (83.33)	67 (80.72)	34 (73.91)	
No	7 (16.67)	16 (19.28)	12 (26.09)	
Use of disposable instead of rechargeable batteries in flashlights/digital cameras				0.16
Yes	18 (42.86)	26 (31.33)	11 (23.91)	
No	24 (57.14)	57 (68.67)	35 (76.09)	
Type of lab coats and patient drapes used in practice				0.048*
Non-reusable	21 (50)	23 (27.71)	17 (36.96)	
Reusable	21 (50)	60 (72.29)	29 (63.04)	
*Recycle*				
Recycling of fixer and developer solutions				0.289
Yes	9 (23.08)	26 (36.62)	12 (27.27)	
No	30 (76.92)	45 (63.38)	32 (72.73)	
Availability of recycling bins in the clinic				0.315
Yes	19 (45.24)	38 (45.78)	27 (58.7)	
No	23 (54.76)	45 (54.22)	19 (41.3)	
Use of sharps disposal service that recycles materials				0.417
Yes	16 (38.1)	30 (36.14)	22 (47.83)	
No	26 (61.9)	53 (63.86)	24 (52.17)	
Frequency of discarding electronic items from clinical practice				0.228
Often	3 (7.14)	9 (10.84)	4 (8.7)	
Sometimes	12 (28.57)	13 (15.66)	13 (28.26)	
Rarely	24 (57.14)	42 (50.6)	22 (47.83)	
Do not discard, we recycle	3 (7.14)	19 (22.89)	7 (15.22)	

Significant *p*-values (≤ 0.05 denoted with “*”) are reported from Chi-square test

Linear regression analysis revealed that having knowledge was significantly associated with higher attitude scores (β = 0.148, *p* = 0.006), with the 95% confidence interval [0.043, 0.253] indicating a robust positive relationship. In contrast, while a positive association was also observed between knowledge and practice scores (β = 1.08), this relationship did not reach statistical significance (*p* = 0.084; 95% CI: [−0.148, 2.308]).

## Discussion

The integration of green dentistry practices is increasingly essential to promote sustainable dental care while minimizing the environmental footprint of dental procedures. Our results found that overall awareness of green dentistry among Pakistani dental professionals is limited, largely due to unfamiliarity with the United Nations’ 2015 Sustainable Development Agenda, which includes ‘sustainable healthcare practices’ [16]. Nevertheless, many participants expressed a strong interest in adopting eco-friendly dentistry, despite facing significant barriers such as financial constraints, particularly among young graduates.

While the majority of participants demonstrated positive attitudes toward sustainable tools like eco-friendly materials and digital radiography, actual implementation remained low. This gap between awareness and practice reflects a broader issue: being informed about sustainable practices does not necessarily translate into their adoption, particularly in developing countries where economic constraints, limited exposure, and difficulties in rural application create significant barriers [17]. These findings are consistent with prior research by Agrasuta et al. [10] and Al Qarni et al. [18], where participants were unfamiliar with the terms “Green Dentistry” or “eco-friendly dentistry” being interchangeable, despite showing interest in sustainable practices. Conversely, studies by Al Shatrat et al. [19] and Pallavi et al. [6] reported higher awareness among their cohorts.

Financial barriers and doubts about the practicality of green initiatives were primary reasons for hesitancy, particularly among post-graduate residents (PGRs), general dental practitioners (GDPs) and dental consultants (DCs). These concerns echo those highlighted by Agrasuta et al. [10] and Kallakuri et al. [20], reinforcing the need for cost-effective, context-appropriate green solutions. Such barriers are especially relevant in low and middle-income countries like Pakistan and India. A recent survey in western India [21] also, revealed implementation to be costly and difficult, despite higher motivation and awareness.

Participants favored tooth-colored restorative materials over amalgam, influenced by the 2013 Minamata Treaty, endorsed and supported by the FDI [22, 23] which aims to phase down mercury use. Despite this awareness, improper amalgam disposal was still reported, posing a serious environmental risk, particularly in developing nations where recycling mercury through amalgam separators is underutilized [24]. The discrepancy likely stems from weak regulatory enforcement and limited recycling infrastructure. Implementing directives similar to the European Union’s ‘Waste Framework Directive’ which mandates appropriate waste management practices among member states, to protect human health and prevent environmental harm [25], could provide a valuable model for policy development in Pakistan.

Digital dentistry practices, particularly digital radiography, were positively received across all participant groups. These findings are consistent with Verma et al. [12], who emphasized the ecological advantages of digital over conventional radiography. Similarly, more than half of the participants (*n* = 24 DCs, *n* = 45 GDPs and *n* = 24 of PGRs) from our survey reported using LED bulbs with motion sensors, aligning with Verma et al. [12] and showing a sign of growing energy consciousness. However, regional inconsistencies were noted. For example, Al-Shatrat et al. [19], found high awareness was high, but low implementation, suggesting that even when knowledge is strong, adoption may be limited by deficiencies in local infrastructure or policy rather than by individual willingness.

Sustainable preferences extended to reusable lab coats, patient drapes, sterilizable instruments, and rechargeable batteries, especially among GDPs and PGRs, supporting prior findings by Verma et al. [12]. Yet, the continued reliance on disposable plastic syringes across all groups (*n* = 35 DCs, *n* = 67 GDPs and *n* = 34 PGRs), likely due to perceived convenience or hygiene concerns, despite their environmental impact, underscores the tension between hygiene concerns and sustainability [20, 26]. This tension mirrors broader regional hesitancy to change established clinical routines.

Our findings also revealed deficiencies in recycling practices. Very few clinics recycle fixer and developer solutions, even though these substances contain hazardous silver. While GDPs exhibited the highest recycling rate, DCs lagged significantly. Similar shortcomings were reported by Pallavi, Al Shatrat and others [6, 19, 27]. Moreover, adoption of sharp disposal services was particularly low among PGRs, indicating the need for improved waste management education and infrastructure.

Lastly, we inquired about digital impressions, digital patient charting facilities, and CAD/CAM adoption. Most participants supported the digitization of patient records, recognizing its role in reducing paper use. The use of CAD/CAM systems showed the highest adoption among DCs, aligning with studies indicating that digital dentistry tools reduce both emissions and material wastage [28–30]. These findings indicate a trend towards future adoption rather than current use.

To date, to the best of our knowledge, this is among the first studies exploring green dentistry awareness, attitudes and practices within Pakistan’s dental community. However, several limitations should be noted. Although a moderate response rate was obtained, the possibility of non-response bias cannot be ignored. The sample was geographically limited, potentially affecting generalizability, which can be overcome with a multi-center study with a larger sample size. The moderate internal consistency for practice-related items likely reflects the heterogeneous nature of green dentistry behaviors such as waste reduction, energy conservation and digital adoption, which do not appear to be strongly intercorrelated. As observed by Striener [31], Cronbach’s α varies with sample characteristics and item diversity, while Tavakol and Dennick [32] highlight that multidimensional constructs can underestimate reliability. Therefore, the moderate α in this study should be seen as an expected outcome of measuring complex practices rather than a methodological weakness. The lower coefficient for practice items likely reflects the heterogeneous nature of the questions, which covered a range of behaviors and contextual factors, as well as the relatively low variability in participant responses. Additionally, as a cross-sectional study, it captures a snapshot in time without assessing changes over time or causality. Focus was primarily on identifying issues rather than proposing solutions. Further research, especially in rural areas, is needed to enhance understanding and provide generalized data. Despite these challenges, this survey provides crucial foundational insights into green dentistry practices among dental professionals nationwide.

To enhance the adoption of sustainable dental practices, several strategies could be considered. Policy-level incentives, such as subsidies for eco-friendly materials and technologies, could alleviate financial barriers. Dental institutions and regulatory bodies might implement mandatory training on green dentistry and introduce sustainability metrics into accreditation systems. These suggestions are based on the fact that improving practices may require additional interventions beyond knowledge alone. Creating public-private partnerships to support infrastructure development, like amalgam recycling and digital integration, as these would also facilitate long-term change.

## Conclusions

The findings of this study indicate a growing interest among dental professionals in adapting environmentally responsible practices. However, a critical gap persists in both knowledge and implementation of sustainable approaches within Pakistan’s dental community. Although some eco-friendly measures are in place, broader adoption remains limited due to financial, infrastructural and educational barriers. To bridge this gap, targeted awareness campaigns and the inclusion of sustainability principles in dental education are essential. Strengthening institutional support and offering policy-level incentives can further facilitate the transition toward more sustainable clinical practices.

## Supplementary Information

Supplementary Material 1. Questionnaire

Supplementary Material 2. Validity of questionnaire

## Data Availability

The data that have been used is confidential.
