# Supplementary material for: Knowledge, attitude, and practice of dental professionals towards green dentistry in Karachi city- a cross-sectional survey
Source: BMC Oral Health. 2025 Nov 24;25:1827. doi: 10.1186/s12903-025-07215-4 (PMC12642028; doi:10.1186/s12903-025-07215-4)
Supplement: Supplementary file 1 — Supplementary Material 1. Questionnaire [file 12903_2025_7215_MOESM1_ESM.docx]

**Annexure I: Questionnaire**

**Background of the study:**

Your response to this survey will help us to evaluate the knowledge, attitude, and practice of Dental professionals towards “Green Dentistry” working in the public and private sectors of Karachi city. The survey is very brief and will only take a few minutes to complete. Your information will remain anonymous and will not be shared in public domain or with people unrelated to the study. Your participation in the survey is completely voluntary and all of your responses will be kept confidential.

**I consent to participate**

1. **Yes**
2. **No**

**Part A: Demographics**

1. Specify your gender:
2. Male
3. Female
4. Age: ____________
5. Graduation year: _________
6. Graduation institute :

1) Private

2) Public

1. What is your academic qualification?

- BDS/DDS
- MSc/MCPS/MS
- MPhil/PhD
- FCPS/FDS/MRCS/American Boards
- Other

1. What is your current academic position?

- Dental graduate
- General Dental Practitioner (GDP)
- Postgraduate resident
- Dental Consultant

1. To which specialty do you belong?

- Oral and maxillofacial surgery
- Prosthodontics
- Orthodontics
- Operative dentistry and Endodontics
- Periodontics
- Pedodontics

1. Which of the following describes your clinical practice?

- Private practice
- Hospital based practice
- Both

**Part B: (I) Green Dentistry (Knowledge assessment):**

Q1): Are you aware of the term “Green dentistry”?

- Yes
- No

Q2): Are you aware of the “4Rs” of Green Dentistry?

- Yes
- No

Q3): Are you aware that the terms "Green dentistry" and "Eco-friendly dentistry" can be used interchangeably?

- Yes
- No

Q4): Are you aware of the term “Sustainable dentistry”?

- Yes
- No

Q5): Are you aware of the agenda by United Nations adopted in 2015 for sustainable development?

- Yes
- No

**II) Attitudes towards green dentistry**

**(Reverse attitude assessment)**

**1. There is nothing a small sector dental practice can do for environmental issues’ (reverse attitude)**

1. Strongly agree
2. Agree
3. Neutral
4. Disagree
5. Strongly Disagree
6. **‘It is difficult to change my current practice to be more ecological’**
7. Strongly agree
8. Agree
9. Neutral
10. Disagree
11. Strongly Disagree
12. **‘Creating a green dental clinic will increase the financial burden on management of the practice’**
13. Strongly agree
14. Agree
15. Neutral
16. Disagree
17. Strongly Disagree
18. **It is difficult to find eco-friendly products for the dental clinic’**
19. Strongly agree
20. Agree
21. Neutral
22. Disagree
23. Strongly Disagree
24. **Recycling waste management is an extra burden on the work of our practice’ (reverse attitude)**
25. Strongly agree
26. Agree
27. Neutral
28. Disagree
29. Strongly Disagree
30. **Sterilized instruments are not as clean as disposable instruments’ (reverse attitude)**
31. Strongly agree
32. Agree
33. Neutral
34. Disagree
35. Strongly Disagree
36. **‘It is not necessary to control water conservation’ (reverse attitude)**
37. Strongly agree
38. Agree
39. Neutral
40. Disagree
41. Strongly Disagree

**(Positive attitude assessment)**

1. **Green practice has many long-term benefits, such as lower energy and water bills’**
2. Strongly agree
3. Agree
4. Neutral
5. Disagree
6. Strongly Disagree
7. **‘Tooth colour-like materials are more environmentally friendly than amalgam**
8. Strongly agree
9. Agree
10. Neutral
11. Disagree
12. Strongly Disagree
13. **Digital radiography is more ecological than conventional techniques’**
14. Strongly agree
15. Agree
16. Neutral
17. Disagree
18. Strongly Disagree
19. **‘Energy management is everyone’s responsibility’**
20. Strongly agree
21. Agree
22. Neutral
23. Disagree
24. Strongly Disagree

**III) Four Rs of Green Dentistry**

Rethink:

Q1). Lighting system is used in your practice

- LED lightbulbs with motion sensors
- Compact fluorescent lamps (CFL)
- Incandescent bulbs
- Other

Q2). Should we go digital to eliminate photochemical waste?

- Yes
- No

Q3). Should Eco-friendly dentistry be universally recommended?

- Yes
- No

Reduce:

Q1): Do you use composite and/or GIC as an alternative to amalgam restorations?

- Yes
- No

Q2). Frequency of amalgam restorations performed in the clinic?

- Always
- Often
- Sometimes
- Rarely
- Never

Q3): Do you have amalgamator in your clinic?

- Yes
- No

Q4) Method of mercury disposal?

- In Sewerage system
- In clinical waste bags or sharp containers
- In waste destined for incineration
- Recycle through Amalgam separator

Re-use:

Q1). Do you use sterilization instruments like trays, film holding devices rather than disposable products?

- Yes
- No

Q2). Do you use disposable plastic syringe instead of reusable glass irrigation syringe in your practice?

- Yes
- No

Q3) Do you use disposable batteries, instead of rechargeable batteries for flashlights and digital camera?

- Yes
- No

Q4). What type of Lab coats and patient drapes is being used in your practice?

- Reusable
- Non-reusable

Recycle:

Q1): Do you recycle the fixer and developer solution?

- Yes
- No

Q2): Do you have recycling bins in your clinic?

- Yes
- No

Q3) Do you use a sharps disposal service that recycles them into building materials?

- Yes
- No

Q4): How often do you discard electronic items from your clinical practice?

- Often
- Sometimes
- Rarely
- Do not discard, we recycle

Digital dentistry:

Q45). Do you have facility of digital impressions in your clinic?

- Yes
- No
- Planning for

Q46). Do you have facility of digital patient charting?

- Yes
- No
- Planning for

Q47) Do your laboratory have CAD/CAM system for the fabrication of prosthesis?

- Yes
- No
- Planning for
